# Dreaming, waking conscious experience, and the resting brain: report of subjective experience as a tool in the cognitive neurosciences

**DOI:** 10.3389/fpsyg.2013.00637

**Published:** 2013-09-23

**Authors:** Erin J. Wamsley

**Affiliations:** Department of Psychiatry, Center for Sleep and Cognition, Harvard Medical School and Beth Israel Deaconess Medical CenterBoston, MA, USA

**Keywords:** sleep, consciousness, dreaming, mentation, memory, cognitive neuroscience, default network

## Abstract

Even when we are ostensibly doing “nothing”—as during states of rest, sleep, and reverie—the brain continues to process information. In resting wakefulness, the mind generates thoughts, plans for the future, and imagines fictitious scenarios. In sleep, when the demands of sensory input are reduced, our experience turns to the thoughts and images we call “dreaming.” Far from being a meaningless distraction, the content of these subjective experiences provides an important and unique source of information about the activities of the resting mind and brain. In both wakefulness and sleep, spontaneous experience combines recent and remote memory fragments into novel scenarios. These conscious experiences may reflect the consolidation of recent memory into long-term storage, an adaptive process that functions to extract general knowledge about the world and adaptively respond to future events. Recent examples from psychology and neuroscience demonstrate that the use of subjective report can provide clues to the function(s) of rest and sleep.

Traditionally, science has studied the human mind by observing how research participants respond to external stimuli. In the course of a day, however, we spend surprisingly little time actively attending to stimuli in our immediate environment. First, during our waking hours, we spend about half of our time thinking about something other than our immediate surroundings—“daydreaming” or “mind-wandering” (Killingsworth and Gilbert, 2010). Beyond this, we spend nearly a third of our lives sleeping. Yet the activity of the brain, as well as our accompanying stream of consciousness, persists throughout all these states of wakefulness and sleep. Here, several recent lines of evidence are described suggesting that dreaming and waking consciousness are not necessarily generated by independent mechanisms, running contrary to centuries of dogma on the fundamental nature of dreaming.

Conscious experience during sleep (i.e., dreaming) has classically been considered a phenomenon entirely distinct from the spontaneous thought and imagery of wakefulness. But to the contrary, emerging evidence suggests that dream experiences may best be conceptualized as a natural extension of waking consciousness, overlapping in both phenomenology and neural mechanism (Wamsley and Stickgold, 2010; Domhoff, 2011; Horikawa et al., 2013). In both resting wakefulness and sleep, the mind/brain is hard at work processing the day's events and concerns—consolidating memory (Plihal and Born, 1997; Mednick et al., 2002; Tucker et al., 2006), integrating new information with our existing knowledge (Tamminen et al., 2010; Lewis and Durrant, 2011), and perhaps even using past experience to plan for the future (Wilhelm et al., 2011). While dreaming and mind wandering are not necessarily functional in and of themselves, as described below, emerging evidence suggests that these conscious experiences are influenced by the neurophysiological functions of the resting and sleeping brain. Thus, the systematic study of subjective experience, across all states of consciousness, may prove crucial to a broader understanding of the brain function during “offline” states.

## Toward a view of dreaming as a normal function of the brain

Scientific progress in understanding dream consciousness has been woefully impeded by classical conceptions of dreams as a “mysterious” and “unknowable” phenomenon resistant to empirical investigation. This view is rooted in traditions that extend back thousands of years and still dominate popular conceptions of dreaming today. Even today, conscious experience during sleep is most often viewed as originating in mechanisms separate from those that generate normal waking thought and perception. In ancient cultures, this was expressed in the view that dreams originate outside the individual as divine messages from gods or spirits. In ancient Greece, for example, citizens suffering from physical ailments would flock to healing temples of the god Asclepius, where they would sleep and receive a divine dream that prescribed (upon interpretation by a priest) a treatment for their condition.

In the early 20th century, the development of psychoanalytic dream theory was ostensibly a break from the superstitious traditions of the past, offering a scientific method of analyzing dreams from a psychological perspective. Yet this paradigm still placed the origin of dreams in a mechanism outside of the traditionally conceived “self”—now dreams came from the mysterious “unconscious mind,” inaccessible during normal wakefulness and rife with sources of pathology. Adding another layer of obscurity is the problematic notion that a dream experience can and should be “interpreted.” Despite thousands of years of dream interpretation, and the proliferation of dream symbol dictionaries on bookstore shelves, there is no systematic empirical evidence that dreams contain symbols to any greater degree than our typical waking thoughts, let alone has there been any empirical support for a particular system to “decode” these symbols. Although Freudian concepts of dreaming have now fallen out of favor in many parts of the psychological community, there has not been a widely accepted new theory of dreaming to take its place.

**Figure 1:**
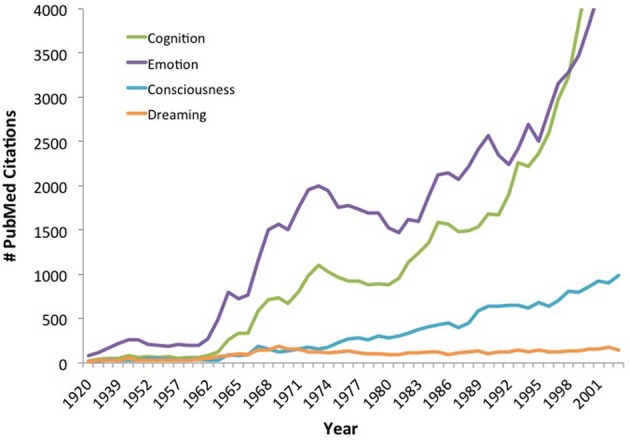
**The cognitive revolution has overlooked sleep.** The cognitive revolution set off a research boom into a variety of long-neglected topics dealing with subjective experience during wakefulness, yet conscious experience during sleep and dreaming have not been targets of a comparable research surge. Counts represent the number of PubMed citations containing the keywords “cognition,” “emotion,” “consciousness” or “dreaming” (within all database fields and MeSH terms) for each year 1920–2003. The search for “dreaming” also included citations containing the MeSH term “dreams.” Both research articles and reviews are included in the citation counts.

Of course, it is not surprising that the question of dream consciousness received little scientific attention during the early-to-mid 20th century, when behaviorism dominated the landscape of psychological research. Following the cognitive revolution, however, as psychologists and neuroscientists moved forward with studying internal states such as emotion, recollection, attention, and consciousness, there was little parallel boom of research into subjective states during sleep (Figure 1). Thus, although cognitive neuroscientists have become increasingly comfortable with using introspective self-report to study wakefulness, conscious experience during dreaming has continued to be treated as a “special case” placed outside the purview of scientific investigation.

Even Alan Hobson's influential neurobiological theories of dreaming (Hobson and McCarley, 1977; Hobson et al., 2000) continued the historical thread of postulating a “special” mechanism for dream generation, non-overlapping with those involved in waking mentation—In this case, the mechanism proposed was activity in the pontine brainstem occurring exclusively during the REM (rapid eye movement) phase of sleep. It is now clear, however, that participants recall dreaming from 50% or more of awakenings outside of REM sleep as well (Foulkes, 1962, 1967; Nielsen, 2000), including even during the deepest stages of slow wave sleep (SWS) (Cavallero et al., 1992; Cicogna et al., 2000). Thus, an eventual cognitive neuroscience of dream consciousness must invoke mechanisms that span across all of the classical “stages” of sleep.

Indeed, in the second half of the 20th century, several theorists began to describe dreaming as a variant of thought and imagery generation that spans the states of wakefulness, REM, and NREM. The seminal work of Calvin Hall, for example, was novel in presenting dream content as a relatively transparent reflection of a dreamers' waking life, rather than a mysterious psychological phenomenon specific to the sleep state (Hall, 1953). Later, the work of Antrobus (Antrobus, 1983, 1991; Reinsel et al., 1992) and Foulkes (1962, 1967) was central in establishing that forms of complex, dreamlike mental activity occurred continuously throughout sleep onset, NREM sleep stages, and even resting wakefulness, suggesting dreaming as a point on a continuum of forms of experience, rather than a phenomenon peculiar to one sleep state. More recent theories have continued to stress how the generation of dream consciousness is related to the neurobiology and cognitive structure of the waking brain (Cicogna and Bosinelli, 2001; Nir and Tononi, 2010; Wamsley and Stickgold, 2010; Perogamvros and Schwartz, 2012).

Several recent lines of evidence continue to suggest that dream consciousness is generated by the same basic neural substrate that supports spontaneous subjective experience during “offline” states of resting *wakefulness*.

## Comparing conscious experience in resting wakefulness and sleep

One reason why dreaming has typically been treated separately from waking conscious experience is that there is assumed to be a bizarre, hallucinatory, and cognitively-deficient phenomenology of dreaming that clearly places it in a separate class of experience. But what is conscious experience really *like* during sleep, and how does this differ from waking thought and imagery? Certainly, experience changes as we move through different states of consciousness—in comparing the form that experience takes during sleep, relative to wakefulness, perhaps the most noteworthy changes are an increase in the vividness of perceptual imagery coupled with attenuated awareness of the outside world (Hobson et al., 2000). Considering the drastic changes in neuromodulation, electrophysiology, and regional brain activation that accompany the onset of sleep, it is certainly not surprising that a corresponding shift in phenomenology would occur. However, despite clear differences between waking mind-wandering and dreaming during sleep, there is little evidence to suggest that conscious experience during dreaming is *generated by a fundamentally different mechanism* than during wakefulness. To the contrary, the available data suggest that dreaming during sleep overlaps in both phenomenology and neural mechanism with spontaneous mentation during offline wakefulness.

Debating whether dream consciousness and waking consciousness are “more similar” or “more different” is a futile enterprise. It is clear that both differences and similarities exist, but tabulating which list has a greater number of items will not necessarily allow us to draw any strong conclusions. Nonetheless, it is important to note that the form and content of conscious experience in wake and sleep do overlap—subjective reports from these different states can, in fact, be so similar as to be indistinguishable. Very vivid, even hallucinatory perceptual imagery, for example, is sometimes described in reports of waking daydreams (Foulkes and Scott, 1973; Foulkes and Fleisher, 1975). Meanwhile, dreams from sleep are not necessarily more “bizarre” than waking mentation. In fact, by one measure, waking fantasy is more “bizarre” than dreaming—the number of sudden “discontinuous” shifts in topic is actually greater in reports of waking fantasy than in dreaming (Wollman and Antrobus, 1986; Reinsel et al., 1992). Conversely, cognition during sleep can be surprisingly logical and coherent, including self-reflection, planning, and focused attention (Kahan et al., 1997; Kahan and LaBerge, 2011).

As an illustrative example of the substantial overlap between waking mentation reports and dreaming, below are two verbal reports from a single participant, one collected from resting wakefulness before sleep and one from Stage 2 NREM sleep:

*I was picturing the dining room at my house. Uh, it's kind of small because we have a very big table in there; there's about 7 or 8 chairs around it and there's another big mirror on the wall, and it's blue – the room is blue. And, um, there's a smaller mirror with a gold frame to the left of the bigger mirror, and you can see into the kitchen from the dining room. There's a little hallway that leads into it*. (Resting Wake)

*I was thinking about… I was in a room and there was someone from my Italian class there, but um, that's it… and there were tables and chairs in the room—kind of set up like desks, but that's it*. (Stage 2 NREM Sleep)

In REM sleep, dream experiences are often longer, more vivid, and more “bizarre” than the examples above. But this is not necessarily the case. Although dream reports from REM are on average longer and more detailed than those collected from NREM sleep, these distributions have substantial overlap (Foulkes, 1962, 1967; Antrobus et al., 1995; Cicogna et al., 1998; Smith et al., 2004; Wamsley et al., 2007). Importantly, many other apparent differences between REM and NREM dreaming (e.g., the amount of “bizarre content,” or the number of events and actions) can be accounted for merely by their greater length (Antrobus, 1983).

## Neurophysiological correlates of subjective experience across states of consciousness

Dreaming has also been considered outside the range of normal brain function because, by all outward appearances, the brain and mind are entirely *“turned off”* during sleep. Indeed, until the 1950's the predominant view of sleep was that of a global state of inactivity, where little or no brain and cognitive processing was occurring. The presence of complex thought and imagery was not easily reconciled with this classical view of the sleeping brain. However, following the advent of all-night EEG recording, and more recently using PET and fMRI neuroimaging, we can now see that the sleeping brain remains very active by several measures. The fast, desynchronized EEG of REM sleep, for example, so resembles that of waking brain activity that this state was initially termed “paradoxical sleep.” Even in the classically “deeper” stages of NREM sleep, neuroimaging studies show that regional metabolic activity is maintained in selected regions (Nofzinger et al., 2002; Peigneux et al., 2004).

**Figure 2:**
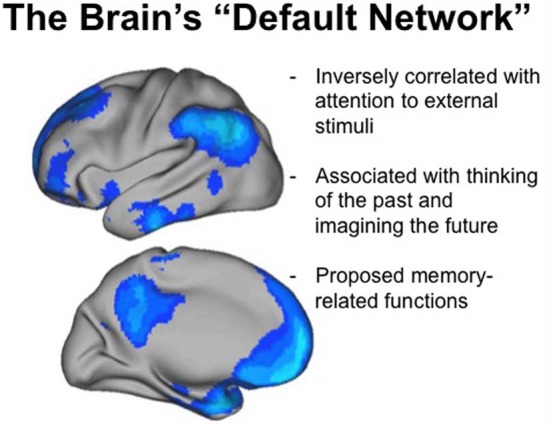
**The “Default Network” of brain function.** Functional imaging studies have identified a consistent network of brain regions that are preferentially active during periods of waking rest, when participants are not engaged in processing external stimuli. Several of these same regions remain relatively active during sleep, including medial frontal and medial temporal networks involved in memory processing. (Adapted with permission from Buckner et al., 2008).

Recent imaging studies have described a consistent pattern of brain activity present during resting wakefulness that overlaps substantially with activity patterns during sleep [the “default network” (Buckner et al., 2008), see Figure 2]. Memory-related regions in the medial temporal and medial frontal regions are amongst the components of this that remain relatively active during both REM and NREM sleep (see Domhoff, 2011 for a recent theoretical paper). During wakefulness, activation of the default network is associated with the generation of conscious thought and imagery (Mason et al., 2007; Andrews-Hanna et al., 2010; Andrews-Hanna, 2012). For example, default network activity is enhanced under conditions of reduced sensory monitoring that increase stimulus-independent thoughts (Andrews-Hanna et al., 2010). Furthermore, individuals reporting more task irrelevant thoughts of the past and future during a resting condition exhibited increased functional connectivity between medial temporal lobe structures and other components of the default network (Andrews-Hanna et al., 2010). Finally, default network activation is also greater in individuals with a strong trait propensity toward daydreaming (Mason et al., 2007). Thus, it appears that there is some structural and functional commonality between the “default mode” of resting wakefulness and patterns of preserved functional activation during sleep. The analogy, however, is not complete. First, parietal regions that form a major component of the waking default network are relatively inactive during both REM and NREM sleep. Second, functional connectivity between default network regions, which is a fundamental feature of how this network is defined during wakefulness, is altered as we enter sleep (Koike et al., 2011; Sämann et al., 2011).

The relationship of default network activity to dreaming during sleep has not yet been directly tested. However, there is some preliminary evidence that particular regions of this network contribute to dream generation. For example, neuropsychologist Mark Solms has described lesions of the ventromedial prefrontal cortex which lead to a reported cessation of dreaming in affected patients (Solms, 2000). Also, recordings of intracranial EEG in epilepsy patients have demonstrated a relationship between hippocampal activity during sleep and the recall of dream experience (Fell et al., 2006).

Thus, despite the apparent inactivity of the sleeping body, and in the face of major neurophysiological changes, regional patterns of brain activity remain partially stable across the transition from resting wakefulness to sleep. During wakefulness, the co-activation of these brain regions is associated with the generation of thought, imagery, and daydreaming. If this functional network is similarly associated with conscious experiences arising during sleep, this would constitute evidence of a shared network for the generation of spontaneous subjective experience, which with some modification, spans across states of consciousness.

## In wake and sleep, spontaneous conscious experience reflects processing past memory and planning for the future

Cognitive neuroscience has now begun to study spontaneous experience during wakefulness in earnest. Emerging data show that far from being a meaningless distraction, so-called “daydreams” provide an important source of information about the activities of the resting brain. One line of this work has been stimulated by interest in the aforementioned “default network”—during periods of quiet rest, activation of the default network (which includes several memory-related regions) is associated with remembering past events, but also with imagining possible future events (Addis et al., 2007; Andrews-Hanna et al., 2010; Andrews-Hanna, 2012). These observations have led to the hypothesis that one function of the brain during rest is to use past memories in constructing simulations of possible futures, enhancing preparedness for future events (Schacter et al., 2007). Also in support of this hypothesis, patients with bilateral damage to the hippocampus (a structure in the medial temporal lobe essential for forming new memories) show not only memory impairments, but are also impaired in their ability to imagine fictitious scenarios and possible futures (Hassabis et al., 2007; Race et al., 2012). Together, with other evidence, these observations suggest that during periods of unoccupied rest, fragments of past experience are reactivated in our minds, and combined into novel imagined scenarios of possible future events.

Several lines of evidence suggest that dream experience may similarly reflect the processing of past memory, as well as the use of memory to simulate future events. First, there is now very strong evidence that sleep is beneficial for the “consolidation” of newly acquired information. For both procedural (Stickgold et al., 2000a; Walker et al., 2002) and declarative (Plihal and Born, 1997; Ellenbogen et al., 2006; Tucker et al., 2006) forms of memory, post-learning sleep has consistently been shown to enhance later memory performance. Furthermore, the processing of memory during sleep appears to be directly reflected in the conscious experience of dreaming. Although past experiences are rarely, if ever, “replayed” in dreams in their complete and original form, nonetheless, a majority of dream reports contain at least one element which can be traced back to a specific recent memory (Fosse et al., 2003). Participants also very often dream about experimental learning tasks (Tauber et al., 1968; Stickgold et al., 2000b; Wamsley et al., 2010a, b; Kusse et al., 2011), and crucially, participants who incorporate learning tasks into their dream content show enhanced memory for the material following sleep (Fiss et al., 1977; De Koninck et al., 1990; Wamsley et al., 2010b). Thus, although the content of dreams is unlikely to be *exclusively* determined by memory-related processes [for example, dreaming may also be influenced by motivational and reward systems (Pennartz et al., 2004; Perogamvros and Schwartz, 2012)], it appears that the consolidation of memory during sleep is one contributor to the construction of dream experience.

Like waking daydreams, there is some preliminary evidence that dreaming during sleep also reflects prospective memory functions, as the brain uses past experience to prepare us for the future. First, we know that sleep does not enhance all past memories equally, but instead selectively strengthens memory for information that is relevant to the future. For example, one recent study found that sleep only enhanced memory when participants expected to be tested the learned information the following morning (Wilhelm et al., 2011). Similarly, sleep preferentially enhances emotional memories (Payne et al., 2008) and memories that participants expect to be rewarded for remembering (Fischer and Born, 2009). Each of these studies illustrates a selective effect of sleep in enhancing memory for information that is *important to an individual*'*s future*. At the same time, it has long been known that the simulation of potential futures forms a substantial part of dream content. For example, Antti Revonsuo's “threat simulation” theory of dreaming builds on evidence that potentially threatening events are played out in imagined scenarios during dreams (Valli and Revonsuo, 2009). As another example, in our own studies using the downhill skiing arcade game *Alpine Racer II*, we found that during a baseline recording night, a small but significant portion (4%) of dream reports anticipated playing *Alpine Racer* the following day, even though participants had never yet seen the game. Taken together, these observations suggest the hypothesis that both spontaneous mentation during relaxed wakefulness and dreaming during sleep may be influenced by the same brain processes: the consolidation of past memory and constructive simulation of future events.

## Spontaneous subjective experience as a tool for cognitive neuroscience

Studies of emotion, memory, decision-making, perception, and consciousness routinely rely on participants' own description of their internal states. Despite the unverifiable nature of such reports, progress in understanding human cognition has immensely benefitted from the use of subjective report as a scientific tool. Why should dreaming be treated any differently? Indeed, open-ended subjective reports from quiet rest and sleep were essential to much of the research described above. Self-report of ongoing conscious experience provides a method of determining whether a specific memory is being reactivated in the resting brain (Wamsley et al., 2010a, b), and offers insight into other brain and cognitive processes which occurring during rest (e.g., future projection, Andrews-Hanna, 2012) and sleep (e.g., reward processing, Perogamvros and Schwartz, 2012).

Importantly, there is as yet no measure of brain activity (e.g., EEG, fMRI, PET) that can demonstrate the reactivation of a *specific* memory trace in the brain during human rest or sleep. For example, while an increase in hippocampal activity during sleep might indicate that memory processing is occurring (e.g., Peigneux et al., 2004), it cannot tell us whether a participant is reactivating the memory of a specific image, word, or thought. Emerging analytic techniques such as multivoxel pattern analysis of the fMRI BOLD signal show immense potential for decoding the neural correlates of recollecting a specific experience (Chadwick et al., 2010), but thus far, their application to defining offline memory reactivation remains in its infancy [though see (Horikawa et al., 2013)]. The conscious retrieval of a recent memory, in contrast, clearly demonstrates that the neural networks encoding that particular memory have been reactivated. Thus, reports of conscious experience offer a valuable source of information about the activity of the resting brain, allowing us to determine *which* memories of everyday waking experience are spontaneously reactivated during offline states of quiet rest and sleep. Regardless of future progress in “decoding” experience based on brain signals, subjective report will continue to provide a valuable window into the cognitive processes occurring during offline states.

Of course, subjective report of experience during sleep does present some unique methodological challenges. As with all subjective report data, we have access only to a participant's *report* of their recent experience, and no objective confirmation is available [although see (Horikawa et al., 2013)]. In dealing with reports of dream experience, this fundamental challenge is compounded by two additional factors—First, verbal reports of experience during sleep are necessarily given retrospectively, elicited only after a participant is awakened and entered a different state of consciousness. Second, memory for dream experiences is poor, relative to memory for waking experience. However, despite the quantitative reduction in recall of experience from sleep, there is no evidence that memory for sleep experiences is inherently less *accurate* than that for waking experience, and thus, there is no reason that such challenges should prevent us from utilizing these valuable data. Just as the challenges of subjective report have not prevented progress in the study of emotion, memory, and consciousness, neither should the limitations of self-report prevent us from studying subjective experience during sleep.

Dreams are not sent to us by the gods, nor are they a disguised message from the unconscious mind. Generated by the same mind and brain that create our waking conscious experience, dreams bear a transparent relationship to waking experience, and provide a useful window into activities of the sleeping brain. Because of this, introspective self-report is a valuable tool for the cognitive neurosciences. Moving into the future, the integration of behavioral, neural, and subjective data will enable us to map the structure, and potential function(s), of spontaneous thought across all states of consciousness.

### Conflict of interest statement

The author declares that the research was conducted in the absence of any commercial or financial relationships that could be construed as a potential conflict of interest.
